# A small and vigorous black hole in the early Universe

**DOI:** 10.1038/s41586-024-07052-5

**Published:** 2024-01-17

**Authors:** Roberto Maiolino, Jan Scholtz, Joris Witstok, Stefano Carniani, Francesco D’Eugenio, Anna de Graaff, Hannah Übler, Sandro Tacchella, Emma Curtis-Lake, Santiago Arribas, Andrew Bunker, Stéphane Charlot, Jacopo Chevallard, Mirko Curti, Tobias J. Looser, Michael V. Maseda, Timothy D. Rawle, Bruno Rodríguez del Pino, Chris J. Willott, Eiichi Egami, Daniel J. Eisenstein, Kevin N. Hainline, Brant Robertson, Christina C. Williams, Christopher N. A. Willmer, William M. Baker, Kristan Boyett, Christa DeCoursey, Andrew C. Fabian, Jakob M. Helton, Zhiyuan Ji, Gareth C. Jones, Nimisha Kumari, Nicolas Laporte, Erica J. Nelson, Michele Perna, Lester Sandles, Irene Shivaei, Fengwu Sun

**Affiliations:** 1 Kavli Institute for Cosmology, University of Cambridge, Cambridge, UK; 2 Cavendish Laboratory - Astrophysics Group, University of Cambridge, Cambridge, UK; 3 Department of Physics and Astronomy, University College London, London, UK; 4 Scuola Normale Superiore, Pisa, Italy; 5 Max-Planck-Institut für Astronomie, Heidelberg, Germany; 6 Centre for Astrophysics Research, Department of Physics, Astronomy and Mathematics, University of Hertfordshire, Hatfield, UK; 7 Centro de Astrobiología (CAB), CSIC–INTA, Madrid, Spain; 8 Department of Physics, University of Oxford, Oxford, UK; 9 Sorbonne Université, CNRS, Paris, France; 10 European Southern Observatory, Garching, Germany; 11 Department of Astronomy, University of Wisconsin-Madison, Madison, WI USA; 12 European Space Agency, Space Telescope Science Institute, Baltimore, MD USA; 13 NRC Herzberg, Victoria, British Columbia Canada; 14 Steward Observatory University of Arizona, Tucson, AZ USA; 15 Center for Astrophysics - Harvard & Smithsonian, Cambridge, MA USA; 16 Department of Astronomy and Astrophysics, University of California, Santa Cruz, Santa Cruz, CA USA; 17 NSF’s National Optical-Infrared Astronomy Research Laboratory, Tucson, AZ USA; 18 School of Physics, University of Melbourne, Parkville, Victoria Australia; 19 ARC Centre of Excellence for All Sky Astrophysics in 3 Dimensions (ASTRO 3D), Melbourne, Victoria Australia; 20 Institute of Astronomy, University of Cambridge, Cambridge, UK; 21 AURA for European Space Agency, Space Telescope Science Institute, Baltimore, MD USA; 22 Department for Astrophysical and Planetary Science, University of Colorado, Boulder, CO USA

**Keywords:** Early universe, Galaxies and clusters

## Abstract

Several theories have been proposed to describe the formation of black hole seeds in the early Universe and to explain the emergence of very massive black holes observed in the first thousand million years after the Big Bang^1–3^. Models consider different seeding and accretion scenarios^4–7^, which require the detection and characterization of black holes in the first few hundred million years after the Big Bang to be validated. Here we present an extensive analysis of the JWST-NIRSpec spectrum of GN-z11, an exceptionally luminous galaxy at *z* = 10.6, revealing the detection of the [Neiv]*λ*2423 and CII*λ1335 transitions (typical of active galactic nuclei), as well as semi-forbidden nebular lines tracing gas densities higher than 10^9^ cm^−3^, typical of the broad line region of active galactic nuclei. These spectral features indicate that GN-z11 hosts an accreting black hole. The spectrum also reveals a deep and blueshifted CIV*λ*1549 absorption trough, tracing an outflow with velocity 800−1,000 km s^−1^, probably driven by the active galactic nucleus. Assuming local virial relations, we derive a black hole mass of $\log ({M}_{{\rm{BH}}}/{M}_{\odot })=6.2\pm 0.3$, accreting at about five times the Eddington rate. These properties are consistent with both heavy seeds scenarios and scenarios considering intermediate and light seeds experiencing episodic super-Eddington phases. Our finding explains the high luminosity of GN-z11 and can also provide an explanation for its exceptionally high nitrogen abundance.

## Abstract (web summary)

An extensive analysis of the JWST-NIRSpec spectrum of GN-z11 shows a supermassive black hole of a few million solar masses in a galaxy 440 million years after the Big Bang.

## Main

GN-z11 was recently observed with JWST. The analysis of the NIRCam images revealed an unresolved nuclear component and a disc-like component with a few 100 parsec (pc) radius^8^. A first NIRSpec spectrum was presented in ref. ^9^, which found it to be consistent with star formation, although the presence of an active galactic nucleus (AGN) was not excluded. Here we explore the latter scenario using a deeper spectrum of GN-z11.

**Fig. 1:**
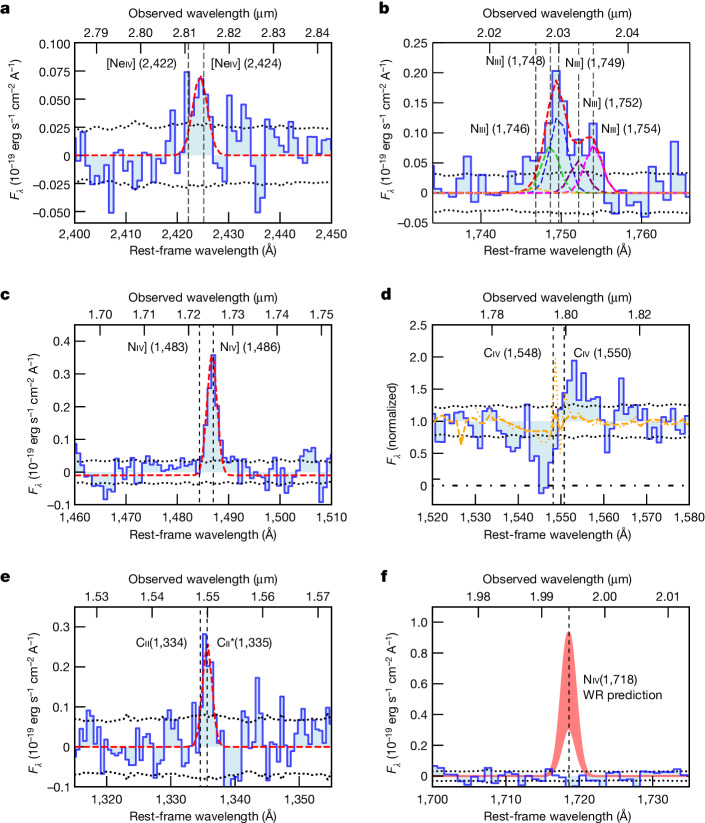
Magnification of the spectra of GN-z11 around specific spectral features of interest, along with their single and multiple Gaussian models (Methods). Dashed lines indicate the rest-frame wavelengths of the lines at *z* = 10.603. **a**, [Neiv]*λλ*2422,2424 doublet; **b**, NIII] multiplet, illustrating the detection of the resolved Niii]*λ*1754 emission; **c**, NIV] doublet, showing the absence of [Niv]*λ*1483 despite the strong Niv]*λ*1486; **d**, CIV blueshifted absorption trough and redshifted resonant emission, compared with the CIV P-Cygni profile observed in low-metallicity, young star-forming galaxies (stack: orange dashed line; most extreme case: orange dotted line), showing inconsistency with the latter. **e**, CII/CII*λλ1334,1335 doublet (seen in emission, without P-Cygni, only in type 1 AGN); **f**, Expected flux of the NIV1718 line in the case that NIV]1486 was associated with WR stars. In **a**–**c**, **e** and **f**, the continuum is subtracted, whereas in **d** the continuum is normalized to one. The grey dotted lines indicate the noise level (1*σ*).

Figure 1a shows the detection of the [Neiv]*λλ*2422,2424 doublet. As NeIV requires photons more energetic than 63.5 eV, this line is an unambiguous AGN tracer^10–12^ and not seen in star-forming galaxies, not even those hosting the Wolf–Rayet (WR) stars^13^.

We also detect CII*λ1335 emission (Fig. 1e). This line is commonly observed in AGN^14–16^. In star-forming galaxies, this line is generally totally undetected; when detected, it is extremely faint and always associated with deep CII*λ*1334 resonant absorption^17^, not seen in GN-z11.

**Fig. 2:**
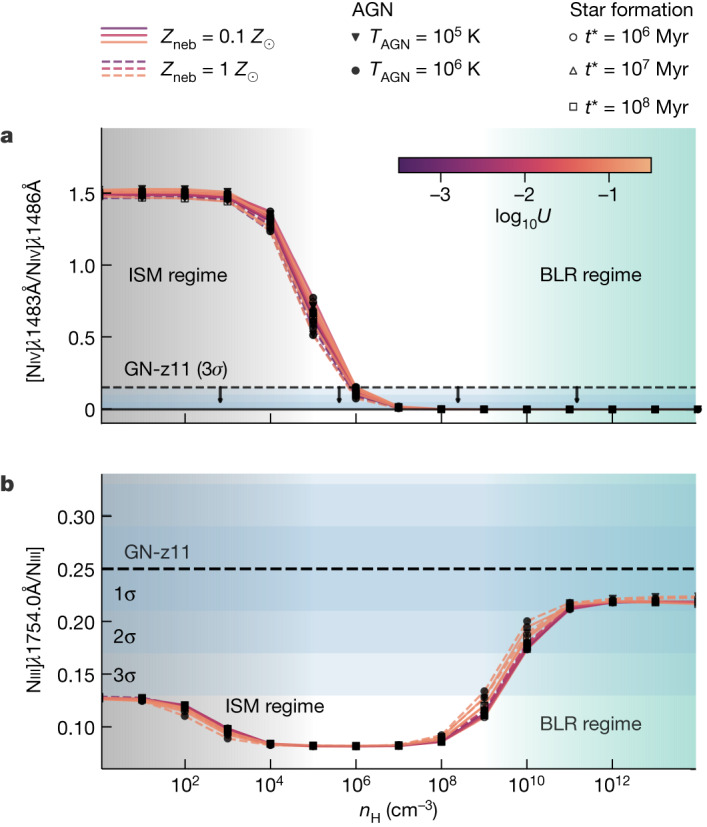
Flux ratios of density-sensitive nitrogen lines as a function of hydrogen gas density, *n*_H_. A large range of Cloudy models (Methods) are compared with the values observed in GN-z11. Models with metal-poor (*Z*_neb_ = 0.1*Z*_⊙_) and metal-rich (*Z*_neb_ = 1*Z*_⊙_) gas are shown with solid lines and dashed lines, respectively (colour-coded according to the ionization parameter *U*), in the scenario in which either an AGN (filled symbols demarcating different black body temperatures for the accretion disc, *T*_AGN_) or stellar populations (open markers for various ages, t_*_) is responsible for the incident radiation field. **a**, [Niv]*λ*1483/Niv]*λ*1486 flux ratio. **b**, Ratio of Niii]*λ*1754 to total flux of the multiplet. The black dashed lines and blue-shaded regions (in decreasing darkness for 1*σ*, 2*σ* and 3*σ* confidence level as indicated) show the observed fractional contribution of Niii]*λ*1754 and upper limit on [Niv]*λ*1483/Niv]*λ*1486 obtained for GN-z11, indicating that the gas emitting these lines has high density (*n*_H_ ≳ 10^9^ cm^−3^ at 3*σ*). The light-green-shaded areas highlight the range of densities typical of the broad line regions (BLRs), whereas the grey-shaded regions highlight the range of densities typical of the ionized interstellar medium (ISM).

The [Niv]*λ*1483, Niv]*λ*1486 doublet is very sensitive to the gas density (and insensitive to ionization parameter, metallicity and shape of the ionizing spectrum). Figure 1c shows the detection of the semi-forbidden Niv]*λ*1486 line (critical density 4.7 × 10^9^ cm^−3^) and the non-detection of the forbidden [Niv]*λ*1483 line (critical density 1.5 × 10^5^ cm^−3^), which indicate densities much higher than 10^5^ cm^−3^. Specifically, the various photoionization models shown in Fig. 2a (see the Methods for details) illustrate that the upper limit on the doublet ratio requires densities greater than about 10^6^ cm^−3^, which are incompatible with the densities of the ionized interstellar medium (ISM) that are typically in the range of 10−10^3^ cm^−3^, and only rarely approach a few times 10^4^ cm^−3^ (ref. ^18^).

Even stronger constraints come from the NIII] multiplet (Fig. 1b). This is contributed primarily by four semi-forbidden lines at 1,748.6 Å, 1,749.7 Å, 1,752.2 Å and 1,754.0 Å (and the much weaker 1,746.8 Å). All of these have high critical densities (>10^9^ cm^−3^), but the 1,748.6 Å and 1,754.0 Å transitions have the highest critical density of 10^10^ cm^−3^ (note that atomic physics requires fixed flux ratios *F*_1754_/*F*_1748_ = 1.05 and *F*_1746_/*F*_1752_ = 0.14; Methods). The 1,754.0 Å line is well resolved compared with the rest of the multiplet, and its intensity is well constrained to be 0.25 ± 0.04 of the total intensity of the multiplet. This high ratio can be achieved only when both the 1,749.7 Å and 1,752.2 Å transitions are suppressed relative to the 1,748.6 Å and 1,754.0 Å because of the very high density. As shown in Fig. 2b, when compared with the expectations from a wide range of photoionization models, the observed ratio requires a density higher than 10^9^ cm^−3^ at 3*σ* (higher than 10^10^ cm^−3^ at 2*σ*). These high densities are completely inconsistent with any HII regions in any star-forming galaxy but are fully in the realm of the broad line regions (BLRs) of AGN, which are characterized by extremely high densities (about 10^9^−10^15^ cm^−3^).

Therefore, the most plausible explanation is that GN-z11 hosts an AGN and that these semi-forbidden lines observed in its spectrum are mostly emitted by the associated BLR.

It may seem puzzling that the spectrum of GN-z11 does not seem to show the typical ‘broad lines’ seen in type 1 quasars and AGN, with widths of thousands km s^−1^. However, the width of the broad lines scales quadratically with the black hole mass, hence in the case of ‘small’ black holes the broadening is expected to be substantially smaller. Moreover, there are classes of type 1 AGN that have broad lines with widths less than 1,000 km s^−1^: these are the so-called Narrow Line Seyfert 1 (NLSy1), the permitted lines of which are broader than their forbidden lines, but not by a large factor, and in many cases reaching a width of only a few 100 km s^−1^ (ref. ^19^), and which are inferred to have small black holes (about 10^6^*M*_⊙_) (ref. ^20^). This seems to be the case of GN-z11, in which the semi-forbidden (NIII], NIV]) and permitted lines (MgII) all have widths between 430 km s^−1^ and 470 km s^−1^, whereas [NeIII] has a significantly narrower width (340 ± 30 km s^−1^), hence coming from the host galaxy (either from HII regions or from the narrow line region of the AGN). We note that the interpretation of some permitted and semi-forbidden lines, such as the Balmer lines and CIII] is made complex by the fact that these are also generally contributed to by the ISM photoionized by star formation in the host galaxy.

The spectrum of GN-z11 also shows a deep (EW_rest_ ≈ 5 Å) and blueshifted absorption trough of the CIV*λλ*1548,1550 doublet (Fig. 1d). Deep CIV absorption is sometimes observed in young stellar populations, but the depth observed in GN-z11 would require high metallicities, typically solar or super-solar^21^. This is in contrast with the metallicity inferred from the nebular lines of GN-z11 (*Z* ≈ 0.1 *Z*_⊙_) (ref. ^9^). To illustrate more quantitatively the inconsistency with the stellar-wind origin, the orange dashed line in Fig. 1d shows the stacked spectrum of local galaxies with metallicity around the value inferred for GN-z11, resampled to the NIRSpec grating resolution: the stellar trough is much shallower than that observed in GN-z11 and with a completely different shape. Apart from the stellar origin, this deep CIV absorption is seen also in lower redshift star-forming galaxies and associated with galactic outflows^22^. However, in these cases the outflow velocities are only of a few 100 km s^−1^ (Methods), whereas for GN-z11 the CIV trough traces a much faster outflow of around 800−1,000 km s^−1^. A more plausible explanation of the deep blueshifted trough of CIV is that GN-z11 is part of the class of broad absorption line (BAL) quasars, which are characterized by deep absorption of blueshifted CIV by up to several thousands km s^−1^. Actually, GN-z11 would fit in the ‘mini-BAL’ category, with velocities between 500 and 2,000 km s^−1^, more common in lower luminosity AGN, or in the ‘narrow’ (approximately 1,000 km s^−1^) absorption line (NAL) quasars category^23^. The spectrum also shows a clear CIV redshifted emission, which is probably tracing the receding component of the outflow. As CIV is a resonant line, this is the counterpart of the redshifted Lyα identified in ref. ^9^ (consistent shift and width).

In sum, the detection of [NeIV] and CII*, the extremely high gas density matching those of the AGN BLRs, and the presence of a deep, blueshifted absorption trough of CIV tracing a high-velocity outflow are all consistent with the scenario in which GN-z11 hosts an accreting black hole, that is, an AGN, specifically what would be called NLSy1 and (mini-)BAL/NAL AGN.

In the Methods, we also discuss other diagnostics, such as the ratio of UV transitions (for example, CIII]/CIV, CIII]/HeII) and the upper limits on high ionization lines (NV*λλ*1238,1242 and [Nev]*λ*3426), are fully consistent with the AGN scenario.

**Extended Data Fig. 1:**
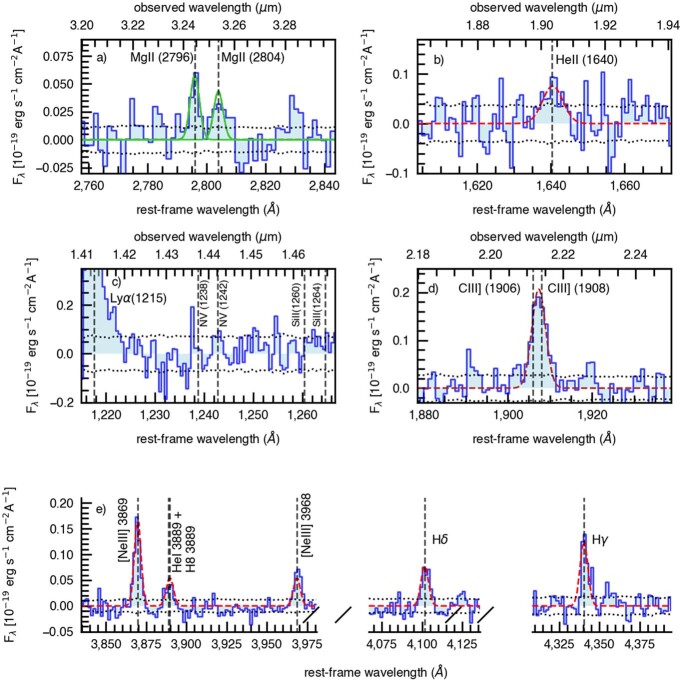
Zoom in on the additional emission lines fitted. **a**) MgII*λ*2796,2804 doublet; **b**) HeII*λ*1640; **c**) Ly*α*, NV*λ*1238,1242 doublet (undetected) and SiII*λ*1260,1264 (undetected), corrected for the Ly*α* damping wing; **d**) Ciii]*λ*1906,1908 doublet. As the doublet is unresolved, the fit turns out degenerate between line width and fluxes of the two components; moreover it is also contributed to by star formation in the host galaxy (see text for details); **e**) [Neiii]*λ*3869 profile compared with the Balmer lines H*δ* and H*γ*. In all panels, the continuum is subtracted. The black dotted lines indicate the 1sigma noise level.

Some works have suggested that GN-z11 may host a population of WR stars^24^. The HeII*λ*1640 line shows a potentially broad profile, as shown in Extended Data Fig. 1b (although the wings are mostly in the noise). This, if confirmed, could come from the inner region of the BLR but could also trace the presence of a WR population. However, various other features are inconsistent with the main contribution from WR stars. Specifically, in the case of WR stars, the NIV*λλ*1483,1486 doublet, if present, is always accompanied by an even stronger NIV*λ*1718 line, with a prominent P-Cygni profile, which is not seen at high confidence in the spectrum of GN-z11 (ref. ^13^) (Fig. 1f); [NeIV] and CII* are never seen associated with WR stars^13^; when present, the NIII] multiplet has a much weaker *λ*1754 component^18^. Therefore, if WR stars are present in GN-z11, then they must co-exist with the AGN and are unlikely to play a dominant part in the excitation of the observed nebular lines.

Assuming local virial relations, the black hole mass can be estimated from the line widths and continuum luminosity. As discussed in the Methods, we estimate a black hole mass of about 1.6 × 10^6^*M*_⊙_. In the Methods, we also discuss potential uncertainties and caveats in the determination of the black hole.

We infer a bolometric luminosity of the AGN of 10^45^ erg s^−1^ (Methods), which is a factor of about 5 higher than the Eddington limit (with an uncertainty of a factor of 2). Super-Eddington accretion is generally inferred for NLSy1s and is one of the scenarios proposed to rapidly grow supermassive black holes in the early Universe^4,25^.

**Fig. 3:**
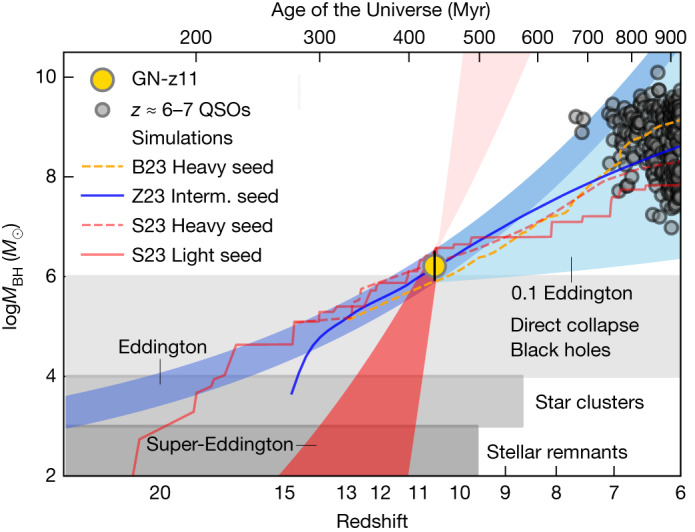
Black hole mass as a function of redshift (on a logarithmic scale) and age of the Universe. The black hole mass inferred for GN-z11 is shown with the large golden symbol. The red-shaded region indicates the evolution expected in the case of super-Eddington accretion at the level inferred for GN-z11. The darker-blue-shaded region shows the black hole mass evolution assuming Eddington-limited accretion, whereas the lighter-blue-shaded region shows the case of evolution in the case of sub-Eddington accretion (between 0.1 and 1 the Eddington rate). The horizontal grey-shaded regions indicate the range of black hole seeds expected by different scenarios. Solid and dashed lines indicate the evolutionary tracks of various simulations and models^7,25,35^ that can reproduce the GN-z11 black hole mass, with different seeding and accretion rate assumptions, as detailed in the Methods. The small grey symbols indicate the black holes measured in quasars (QSOs) at *z* ≈ 6–7.5 (refs. ^1,2^) (whose representative 1*σ* error bar is shown in the top left), most of which can originate from a progenitor such as the black hole in GN-z11.

Figure 3 shows how the black hole mass in GN-z11 would have evolved at earlier cosmic epochs if accreting at the Eddington rate, or at the super-Eddington rate estimated at the time of observation. For comparison, the grey-shaded areas show the range of possible black hole seeds scenarios: black holes resulting from the direct collapse of primordial clouds into seeds with masses in the range of about 10^4^−10^6^*M*_⊙_, the so-called direct collapse black holes (DCBHs); rapid merging of stars and black holes in dense, nuclear star clusters; accretion onto Population III black hole seeds or even normal stellar remnants^1,3–6^. Many of these semi-analytical models and cosmological simulations could reproduce the mass of GN-z11 at *z* = 10.6 (refs. ^7,25–27^). The solid and dashed lines show the evolutionary tracks for some of them (described more extensively in the Methods). These can be broadly divided in models assuming heavy seeds (DCBH), whose accretion is limited to the Eddington rate, and intermediate mass (stellar clusters) or light (stellar remnants) seeds experiencing episodes of super-Eddington accretion. It is interesting to also note that GN-z11 evolving at sub-Eddington rate can easily result into the supermassive black holes (10^7^−10^9^*M*_⊙_) observed in quasars at *z* = 6–7, as predicted by many models.

However, it is possible that the local or low-*z* scaling relations do not apply for AGN at such early epochs. If we disregard the local virial relations and instead assume that the black hole in GN-z11 is accreting at the Eddington rate, then the black hole mass would be 10^7^*M*_⊙_. A black hole with this mass is more difficult to account for, but achievable in models assuming heavy seeds and episodes of super-Eddington accretion^7,26,27^.

**Fig. 4:**
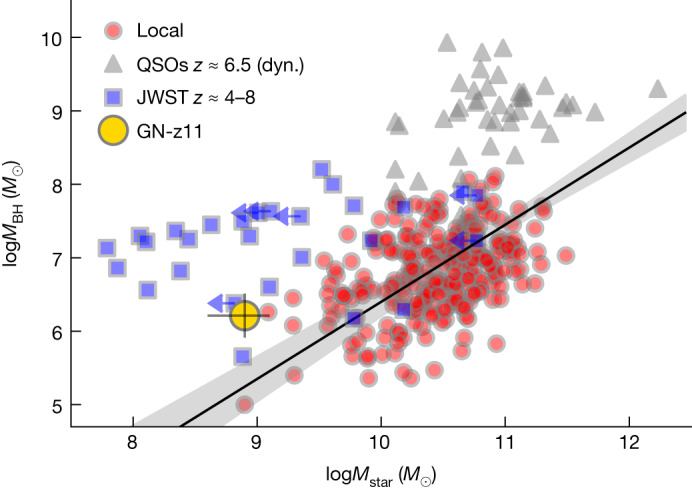
Black hole versus stellar mass diagram. The location of GN-z11 (large golden symbol) is compared with local galaxies as indicated by the small red symbols and their best-fit relation (black solid line and uncertainty traced by the grey-shaded region)^36^. The grey symbols show the values estimated for quasars (QSOs) at *z* ≈ 6−7 (ref. ^37^), although in these cases the galaxy mass is inferred from dynamical tracers. The blue symbols are AGN at *z* > 4 for which the black hole and galaxy stellar mass has been measured with JWST data (Methods) using the same calibration as in ref. ^36^ for consistency.

Taking the stellar mass of the extended disk-like component measured in ref. ^8^ (M_*_ = 8 × 10^8^*M*_⊙_), it is possible to locate GN-z11 on the *M*_BH_–*M*_star_ relation. As shown in Fig. 4, GN-z11 is placed above the local relation, although marginally consistent within the scatter. An early evolution above the local *M*_BH_–*M*_star_ relation is what is expected from models invoking DCBHs and/or super-Eddington accretion^3,4,25^.

We note that the exceptionally high nitrogen abundance inferred for GN-z11 (specifically, high N/O)^9^ becomes much less problematic in the AGN scenario. To begin with, several ‘nitrogen-loud’ AGN have already been found both at low and high redshifts (including NLSy1)^28–31^. So GN-z11 is not a very peculiar system in this context. Second, the mass of the BLR in AGN is very small^32^:

$${M}_{{\rm{BLR}}}=1.8\left(\frac{{L}_{{\rm{H}}\gamma }}{1{0}^{42}\,{\rm{erg}}\,{{\rm{s}}}^{-1}}\right){\left(\frac{n}{1{0}^{10}{{\rm{cm}}}^{-3}}\right)}^{-1}\,{M}_{\odot }$$

Specifically, the H*γ* luminosity observed in GN-z11 (*L*_H*γ*_ = 1.7 × 10^42^ erg s^−1^, even assuming the extreme case it is not contributed by the NLR and HII regions) implies a mass of the BLR of only a few solar masses. It would take just one or two supernovae to enrich such a small mass to solar or super-solar metallicity, especially within the small physical region associated with the BLR (about 10^−2^ pc for GN-z11) (ref. ^33^). These could be supernovae from supermassive stellar progenitors, with high nitrogen yields^34^. However, even without invoking exotic scenarios, given the accelerated metal enrichment of such a small, central region, it is also possible that secondary, recycled nitrogen production can occur within a timescale of a few tens Myr (especially given the very fast cooling times at such high densities, which allow star formation to quickly occur out of cooled SN ejecta).

We finally discuss our results within the context of the recent JWST findings of an excess of exceptionally luminous galaxies at high redshift. GN-z11 is one of the first of such hyperluminous galaxies at high-*z* to be spectroscopically confirmed, and for which such a detailed spectroscopic analysis has been feasible. The AGN scenario revealed by our analysis provides a natural explanation for the exceptional luminosity of GN-z11. If this is representative of the broader class of luminous galaxies discovered at high-*z*, then it would greatly alleviate the tension with models and simulations.

## Methods

### Observations and data processing

The data presented in this paper are part of the JADES survey^38^ and, specifically, obtained through programme ID 1181 (principal investigator D. Eisenstein). GN-z11 was observed in two epochs: the first one on UT 5 and 7 February 2023 and the second one on UT 4 and 5 May 2023. The February observations were already presented in ref. ^9^. We refer to that paper for a detailed description. Briefly, the spectroscopic data were obtained with four different configurations of the NIRSpec micro-shutter array (MSA)^39–41^, using a three-shutter nodding pattern. Four different dispersers were used to cover the 0.6–5.3 μm wavelength range: the low-resolution prism mode (exposure time of 6,200 s, per configuration), and three medium-resolution gratings (3,100 s each, per configuration), which provide a nominal spectral resolution of *R* ≈ 1,000 for a uniformly illuminated slit^39^. However, the highly compact light profile of GN-z11, with respect to the width of the slit, results in a substantially higher effective resolution. To estimate the effective resolution, we forward model the morphology of GN-z11 through the NIRSpec instrument for the grating dispersers, finding that the resolution ranges between 1,100 and 2,100. Four MSA configurations were used (two pointing and two dither positions). The May observations were similar, but in this case they consisted of three consecutive dithers (with three different MSA configurations), each with three nods, resulting in an on-source exposure of 2.7 h for the prism, each of the three medium-resolution gratings and also with the high-resolution grating G395H/290LP. Unfortunately, at the location of GN-z11 on the MSA the latter spectrum is heavily truncated at wavelengths longer than 4.1 μm, hence all strong optical emission lines are not observed with this grating.

By combining the two sets of observations, the total exposure time is 9.6 h with the prism and 6.15 h with each of the medium-resolution gratings.

The data processing is also described in ref. ^9^, and we refer to that paper for a detailed discussion. Here we only mention that we used the pipeline developed by the ESA NIRSpec Science Operations Team and the NIRSpec GTO Team. Most of the processing steps in the pipeline adopt the same algorithms used in the JWST Science Calibration Pipeline^42^. Different from the official pipeline, the final one-dimensional (1D) combined spectra are obtained by combining the 1D individual spectra rather than performing the extraction process in the combined two-dimensional spectra. This step guarantees that the final 1D spectra are well flux calibrated for slit losses. In the combination process, we also applied a 3*σ*-clipping algorithm and excluded bad pixels based on the data quality files provided by the pipeline. The extraction of 1D spectra in the individual exposures is also optimized on the basis of science. In this paper, we adopt a three-pixels (0.3″) extraction along the slit, as it improves the signal-to-noise ratio (S/N) of the spectrum (for point sources). Finally, the GTO pipeline provides spectra beyond the nominal wavelength range for the spectral configuration G140M/F070LP by taking into account the transmission filter throughput in the flux calibration processing step. The extended spectra cover the wavelength range of 1.27–1.84 μm.

Moreover, here we combine the grating spectra in their overlapping ranges, which increases the S/N in those regions. The combined spectra in these regions were resampled to 8 Å around the NIV doublet, not to affect resolution, and to 12 Å around the CIV, as in this case higher S/N is required on the continuum to properly trace the CIV absorption.

In the paper, we adopt the flat ΛCDM cosmology from Planck18 with *H*_0_ = 67.4 km s^−1^ Mpc^−1^ and *Ω*_m_ = 0.315 (ref. ^43^).

### Emission line fitting

The emission lines were fitted using single or multiple Gaussian lines and a simple power law for continuum subtraction. The best-fit parameters for the continuum and Gaussian components were found using the MCMC (Markov chain Monte Carlo) algorithm to estimate the uncertainties. For the purposes of this paper, each line in the rest-frame UV was fitted independently, except for doublets or multiplets, whose line widths were forced to the same value (but see discussion below for the CIII] doublet) and the relative wavelength separation of the doublet and multiplet was forced to the nominal rest-frame wavelength. The absolute velocity of each line (or group of lines in the case of doublets and multiplets) was not constrained to the exact redshift given in ref. ^9^, to allow for small wavelength calibration uncertainties associated with the positional uncertainties of the target within the shutter. (The uncertainties in the target acquisition may result in the target being offset by up to about 0.05″ relative to the nominal position, which is used by the pipeline for the wavelength solution. This unknown offset would introduce a wavelength offset by up to 0.5 spectral pixel, which corresponds to a different velocity offset (given the wavelength-dependent resolution) in different regions of the spectrum.) (Jakobsen, personal communication) and also to allow for small velocity shifts between different lines, which are common in AGN, and especially in the BLR. We restricted our fitting to the lines of interest for this paper.

Extended Data Table 1 provides a list of the fitted emission line widths and fluxes and Extended Data Fig. 1 shows the additional fitted lines not shown in the main text.

In the case of the NIII] multiplet, the 1,748.6 Å and 1,754.0 Å transitions come from the same upper level, hence their flux ratio is fixed by the associated Einstein coefficients, specifically *F*_1754_/*F*_1748_ = 1.05. Similarly, 1,746.8 Å and 1,752.2 Å come from the same upper level and their flux ratio is fixed to *F*_1746_/*F*_1752_ = 0.14. The inferred line widths are deconvolved from the line spread function as inferred for the GN-z11 light profile.

For the CIII]1906,1908 doublet, it is not possible to resolve the two components; attempting to fit it with two components makes the fit degenerate between the width and intensity of the two components. The additional caveat of this CIII] doublet is that it is also commonly seen in normal star-forming galaxies and in the NLR of AGN, so it can also have a contribution from the host galaxy, as for the [NeIII] emission. In ref. ^44^, the authors use IFS spectroscopy to reveal that the CIII] emission is resolved on scales of several 100 pc. As a consequence, we do not include the (spectrally) unresolved CIII] in our analysis, as it does not provide constraints on either the BLR or the host galaxy. In Extended Data Table 1, we report the total flux and width using a single Gaussian. However, for the sake of completeness, we report that by fitting two components with separate full-width at half maximum (FWHM), accounting for the NLR and BLR, gives Ciii]*λ*1906/*λ*1908 of ${0.62}_{-0.37}^{+1.00}$ for the narrow components, with FWHMs of 314 ± 120 km s^−1^ (consistent with the [NeIII] width), and 560 ± 80 km s^−1^ for the Ciii]*λ*1908 broad component (consistent with the NIV] width).

The [OII]3726,3729 doublet would potentially be an additional forbidden line, detected in the observed wavelength range, which could be used to constrain the velocity dispersion in the host galaxy. However, unfortunately, the doublet is unresolved. Attempting to fit it (by forcing the two components to have the same width) results in a FWHM of 365 ± 55 km s^−1^ and a flux ratio of ${0.62}_{-0.21}^{+0.31}$.

### CIV absorption and emission

In this section, we provide some additional details on the CIV absorption. As mentioned in the text, CIV P-Cygni profiles with a significant CIV blueshifted trough are seen associated with atmospheres of young, hot stars. Yet, the depth of this feature is a strong function of metallicity^21^, and the deep trough observed in GN-z11 would require stars with solar or even super-solar metallicities, completely inconsistent with the much lower metallicity inferred for GN-z11. To illustrate the inconsistency with the pure stellar origin, we have stacked 11 UV spectra from the CLASSY HST survey^17^, with metallicity around the value inferred for GN-z11 in ref. ^9^ (*Z* = 0.1 *Z*_⊙_), specifically 7.4 < 12 + log(*O*/*H*) < 7.9. We were conservative by excluding galaxies with strong CIV emission. We also excluded one WR galaxy, as we discuss that the spectrum cannot be dominated by WR stars (see main text and section ‘The WR scenario’). The continuum of the spectra was normalized to one by using a simple linear fit in the spectral ranges 1,410–1,480 Å and 1,560–1,600 Å, consistent with the analysis of the spectrum of GN-z11 in the same spectral region (Fig. 1d). The resulting stacked spectrum is shown with a dashed, orange line in Fig. 1d and illustrates inconsistency with the trough seen in GN-z11. To be conservative, in Fig. 1d, we also show the case of the most extreme spectrum among the 11 selected, the one with the deepest CIV absorption. Although the wings of the stellar winds can extend out to 2,000 km s^−1^, the profile and depth observed trough at these metallicities is inconsistent with the observed trough in GN-z11 at 5*σ*.

The blueshifted CIV trough (and redshifted emission), therefore, is not a P-Cygni feature associated with stellar (atmosphere) winds. Rather, it is tracing a galactic outflow, as observed in lower redshift starbursts^22^ and in (mini-)BAL/NAL AGN^23,45–49^. The determination of the velocity requires knowledge of the exact wavelength of the redshifted, rest-frame CIV transition. Unfortunately, there are small wavelength uncertainties associated with each grating because of the uncertainties of the location of the sources within the shutter, as discussed above. In this specific case, we calibrate the velocity shift based on the NIV line, which is in the same gratings and has a similar ionization potential as CIV. The outflow velocity is also subject to different definitions. The centroid of the trough relative to the mean of the two CIV transitions gives a velocity of –790 km s^−1^. If we consider the blue edge of the trough relative to the bluest of the two transitions (CIV*λ*1548.19), then we obtain a velocity of –1,040 km s^−1^. These velocities are significantly higher than those inferred from the CIV absorption in starburst-driven outflows^22,50^, but in the range of BAL quasars that can span from 500 km s^−1^ to several thousands km s^−1^ (refs. ^23,45–49,51,52^).

The classification boundary between mini-BAL and NAL AGN is not sharp, with different authors giving different definitions in terms of width and/or blueshift of the absorption^23,45–49^. Here we simply give a generic classification as mini-BAL/NAL without aiming at a more specific category.

It should be noted that some past works have reported some rare starburst galaxies showing outflows with high velocities, even in excess of 1,000 km s^−1^ (refs. ^53–56^). However, these outflows are traced by lower ionization transitions (MgII absorption and [OIII] emission). More importantly, a close inspection of those cases reveals that each of them shows some AGN signature ([NeV] emission and/or broad MgII emission and/or broad Hβ emission and/or X-ray emission and/or located in the AGN or composite region of diagnostic diagrams). Therefore, although the AGN contribution to the bolometric luminosity of these galaxies may be arguable (also taking into account the variable nature of AGN), it is likely that the high-velocity outflows seen in these rare cases are actually driven by the AGN that they host.

Finally, it should be noted that the CIV absorption trough goes nearly to zero (as in many BAL quasars), which implies the total covering factor of the emitting source by the outflowing ionized gas along our line of sight. However, the errors leave scope for a contribution of 30% of the emission potentially not covered by the CIV absorption, which can be associated with the extended host galaxy. Yet, if higher S/N data confirm the CIV trough going to zero, this would imply that the outflow has an extent covering also the host galaxy, that is, about 400 pc, which would be fully consistent with recent findings of BAL outflows extending on scales of up to several kpc (refs. ^49,57–59^).

Given that CIV is a resonant line, the observed redshifted emission is also tracing the CIV counterpart of the redshifted Lyα emission seen in ref. ^9^—that is, the receding side of the outflow.

We finally note that the spectrum between Lyα and the NV doublet shows the tentative signature of an NV blueshifted trough (Extended Data Fig. 1d), which would be associated with the highly ionized outflow, but it requires additional data to be confirmed.

### Constraints from other emission lines and diagnostics

Although the paper focuses on a few lines discussed in the main text, in this section we also discuss other emission lines that have either lower S/N, more severe blending or whose upper (or lower) limits give line ratios that are fully consistent with the AGN scenario.

#### MgII and CIII]

The MgII2796,2804 doublet is well resolved with the grating and in principle a good tracer of gas density in the range between 10^9^ cm^−3^ and 10^14^ cm^−3^. However, the observed ratio, $1.3{6}_{-0.42}^{+0.67}$, is so uncertain to be consistent both with the low-density regime (ratio of around 1) and the high-density regime (ratio of about 2). Moreover, even if additional data allows constraining the MgII doublet ratio more tightly, these are resonant transitions, which are, therefore, strongly sensitive to the optical depth and radiative transfer effects^60^.

The Ciii]*λλ*1907,1909 doublet would also be a good density tracer, as the ratio of its two components is primarily sensitive to the gas density and changes strongly between 10^4^ cm^−3^ and 10^6^ cm^−3^ (with the blue component *λ*1907 going to zero at high densities), similar to the NIV] doublet. However, as discussed above, the two components are unresolved with the grating, and we cannot obtain reliable constraints on the gas density or on the line widths. More importantly, CIII] emission is commonly seen also in star-forming galaxies and in the NLR of AGN, so it may partially come also from the low-density ISM of the host galaxy, as is the case for [NeIII]. As already mentioned, recent IFS observations show CIII] to be resolved on scales of several 100 pc (ref. ^44^). It is interesting that when fitted with narrow and broad components, as discussed in the previous section, the narrow component gives widths formally consistent with the [NeIII], whereas the broad component is consistent with the NIV width.

#### NV and NeV

Additional transitions from species requiring ionizing photon energy higher than about 60 eV, such as NV and NeV (in addition to NeIV seen in GN-z11), are often seen as evidence for the presence of an AGN. Yet, conversely, their absence should not be necessarily seen as evidence for the absence of an AGN, as often these lines are weak even in AGN and remain undetected if the S/N is not high enough^61–63^. Moreover, the intensity of these lines varies strongly from case to case.

**Extended Data Fig. 2:**
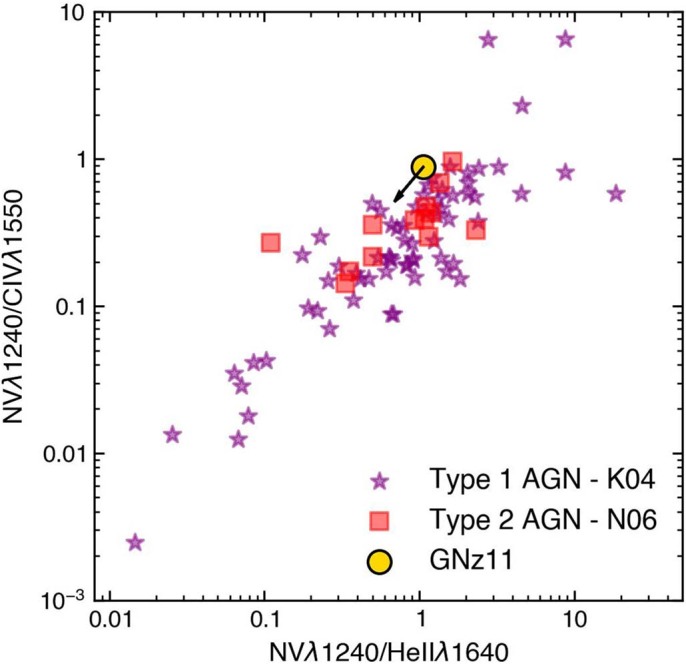
NV/CIV versus NV/HeII flux ratio diagram. GN-z11 (golden circle) is compared with the ratios observed for the broad lines of type 1 AGN (purple stars^61^), and for the NLR of type 2 AGN (red squares^62^), illustrating that the non-detection of NV for GN-z11 is not constraining and consistent with the AGN scenario.

With the prism it is not possible to assess the presence of NV because it is blended with Lyα and its damping wing. Regarding the gratings, the G140M band, in which NV is redshifted, is the least sensitive of the three medium-resolution spectra. Although there is a hint of the NV doublet (Extended Data Fig. 1a 2*σ* integrated signal) we obviously do not quote it as a tentative detection. The inferred upper limit on the NV emission is not very constraining, but the important aspect in the context of this paper is that it is still fully consistent with the presence of an AGN. We demonstrate this in Extended Data Fig. 2, in which the upper limits on the NV/CIV and NV/HeII ratios for GN-z11 are compared with a sample of the broad lines in type 1 AGN^61^ and also with a sample of the NLR in type 2 AGN^62^, and illustrating that the non-detection of NV is fully consistent with the AGN scenario.

It is also interesting to compare NV with NIV, as this ratio is not dependent on the nitrogen abundance, although NIV is detected (or reported) less frequently in AGN. In the well-studied type 1.8 AGN at *z* = 5.5, GS-3073 (refs. ^16,30,64^), the NV is five times fainter than NIV, which would be totally undetected in our spectrum. In the type 1 quasars explored in ref. ^65^, the NIV broad line is very strong, whereas NV is undetected, with an upper limit that is about 10 times lower than the NIV flux.

NeV is also not detected, neither in the grating nor in the prism spectrum. The upper limit on the NeV/NeIII ratio is about 0.2. However, in ref. ^63^, the authors have shown that AGN models can have NeV/NeIII as low as 10^−2^−10^−4^. Hence the non-detection of NeV is also not constraining about the presence of an AGN.

Finally, we note that AGN accreting at super-Eddington rates have a lower energy cutoff, and hence are less likely to emit hard photons that can produce highly ionized species, such as NV and NeV.

#### HeII and CIV

HeII is detected in the prism and, more marginally, in the grating (Extended Data Fig. 1).

As already discussed, CIV is detected in the grating, but with a P-Cygni profile, hence its flux is a lower limit because of self-absorption.

**Extended Data Fig. 3:**
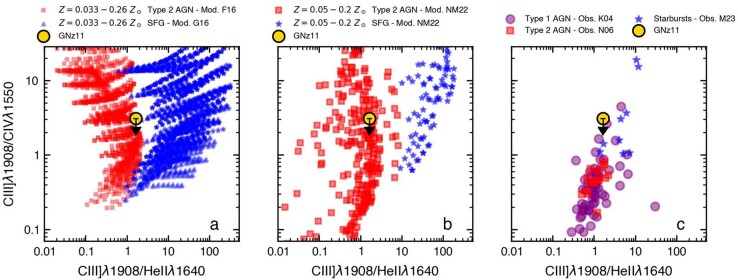
CIII]/CIV versus CIII]/HeII flux ratio diagrams. GN-z11 (golden circle) is compared with: a) the AGN-NLR models (red squares) by^10^ and SF galaxies (blue stars) by^66^ (left) and with b) the AGN-NLR models (red squares) and SF models (blue triangles) by^67^ (centre). All models have been chosen in a low metallicity range, around the value inferred by^9^ for GN-z11. c) Comparison of GN-z11 with the ratios *observed* for the broad lines of type 1 AGN (purple circles^61^), narrow lines of type 2 AGN (red squares^62^), and starburst galaxies (blue stars^18^).

The interpretation of these limits using photoionization models is very much model-dependent. We illustrate this in Extended Data Fig. 3a,b. Specifically, Extended Data Fig. 3a, as in ref. ^9^, shows the location of GN-z11 on the CIII]/CIV versus HeII/CIII] diagram and in which the red-squared and blue-starred symbols show the location of models from refs. ^10,66^ for the NLR of AGNs and for star-forming galaxies, respectively, and in a range of about ±0.3 (see legend) dex of the metallicity inferred in ref. ^9^ for GN-z11. GN-z11 can be consistent with both AGN and star-forming models.

Extended Data Fig. 3b shows the same diagram in which we instead plot the models from ref. ^67^, in the same (low) metallicity range for both AGN and SF galaxies. In this case, GN-z11 is much more consistent with the AGN models and inconsistent with the models for star-forming galaxies.

Yet, if the permitted and semi-forbidden lines of GN-z11 are coming from the BLR, as argued in this paper, then neither of the models above actually apply, as they are developed for the low-density environments of the NLR and HII regions. It is, therefore, more instructive to compare with the line ratios observed in the BLR of type 1 AGN. These are taken from the compilation of ref. ^61^ and shown with purple circles in Extended Data Fig. 3c. The line ratios observed in GN-z11 are fully consistent with the broad lines of type 1 AGN. For completeness, in the same panel we also plot the ratios observed for the NLR of type 2 AGN, compiled in ref. ^62^ (mostly overlapping with the ratios observed for the broad lines), and the star-forming galaxies from the CLASSY survey^18^.

### The WR scenario

In this section, we discuss the scenario recently proposed that GN-z11 may be similar to local WR galaxies^24^.

The HeII marginal detection shows a potentially broad profile (about 10,00 km s^−1^, although the broad wings are mostly in the noise), which may be associated with the inner BLR, but also may resemble the broad HeII profile characteristic of WR stars. Therefore, there might be a contribution from WR stars and, specifically, WN stars, given the strong nitrogen lines.

However, there are various spectral features that cannot be accounted for in the WN scenario.

WN stars are also characterized by very strong NIV*λ*1718 resonant emission, stronger than the NIV*λ*1486, and typically with a prominent P-Cygni profile^13^. In GN-z11, despite the very strong NIV*λ*1486, there is no trace of the NIV*λ*1718 line. Figure 1f shows the spectrum of GN-z11 at the expected location of NIV*λ*1718 and in which the shaded red region shows the expected intensity of the line, based on the strength of the NIV*λ*1486 line. The GN-z11 spectrum is totally inconsistent with the presence of the NIV*λ*1718 WR signature.

Furthermore, neither [Neiv]*λ*2424 nor CII*λ1335 are ever seen associated with the WR population^13^.

Finally, even if WN show prominent NIII] emission, the strength of the *λ*1754 component of the multiplet is much fainter in WR galaxies such as Mrk966 (ref. ^17^) and consistent with densities typical of the ISM.

In sum, although WR stars might be present in GN-z11, they are unlikely to dominate the excitation of most nebular lines.

Extended Data Table 2 summarizes more schematically the observational features consistent or inconsistent with the AGN scenario, the WR scenario and a compact starburst without WR stars.

### Photoionization modelling

We used the Cloudy photoionization code^68^ to explore the effect of varying physical conditions on some emission line ratios constrained by JWST/NIRSpec. The primary goal is to explore the ratios of emission lines within a given doublet or multiplet, hence lines of the same ion (specifically NIII and NIV) that are effectively insensitive to the chemical abundance and ionization parameter, while sensitive to density and only with secondary dependence on temperature. For this reason, the details of the photoionization models are not as important as when exploring other line ratios. We considered a nebula of constant pressure in plane-parallel geometry. However, we have verified that other scenarios, such as a cloud with constant density, do not affect our findings. For completeness, we considered both AGN and stellar templates for the shape of the incident radiation field. Its normalization is set by the ionization parameter, defined as *U* ≡ *Φ*_H_/(*n*_H_*c*), where *Φ*_H_ is the surface flux of hydrogen-ionizing photons at the illuminated face of the nebula, *n*_H_ is the number density of hydrogen and *c* is the speed of light. The hydrogen density and ionization parameter were varied in logarithmic steps of 1, respectively from *n*_H_ = 1 cm^−3^ up to *n*_H_ = 10^14^ cm^−3^, and starting at log_10_*U* = −3 and ending at log_10_*U* = −1 (refs. ^10,66,67^).

In the AGN scenario, we adopted the multi-component continuum template implemented in Cloudy, consisting of a black body and a power law, varying the black body temperature (*T*_AGN_ = 10^6^ K and 10^6^ K) while fixing the power-law slope to *α* = −1.4 (note that this is the slope underlying the black body at energies above the Ly-edge) and leaving other optional parameters as default. For the AGN models, we considered gas-phase metallicities of *Z*_neb_ = 0.1 *Z*_⊙_ and *Z*_neb_ = 1 *Z*_⊙_. By contrast, the star-forming models are restricted to *Z*_neb_ = 0.1 Z_⊙_, as the hard ionizing spectra of metal-poor stars are essential to form sufficient triply ionized nitrogen (requiring 47.5 eV), whose presence in GN-z11 is evidenced by the strong NIV emission (EW_NIV 1486_ = 9.0 ± 1.1 Å; ref. ^9^), whereas metal-rich stars would not produce enough hard ionizing photons to make the NIV line visible. In the star-formation scenario, we used stellar population synthesis models, including binary stars generated by bpass v.2.1 (ref. ^69^) for a single burst of star formation (with varying ages, t_*_/Myr ∈ {1, 10, 100}), assuming the same metallicity as the gas (that is, Z_*_ = *Z*_neb_ = 0.1*Z*_⊙_) and an IMF^70^ that ranges in stellar mass from 1*M*_⊙_ to 100*M*_⊙_. Both in the AGN and star-formation cases, calculations are run until a neutral hydrogen column density of *N*_HI_ = 10^21^ cm^−2^ is reached to ensure that in all models the nebula is matter bounded; we note, however, that the highly ionized nitrogen lines are produced in the very inner part of the cloud, such that the boundary conditions do not significantly affect our results. In total, this results in a parameter grid of 15 different densities, 3 ionization parameters, 3 temperatures or stellar ages, 2 or 1 metallicities for the AGN and star-formation models, respectively, or a total of 15 × 3 × (3 × 2 + 3 × 1) = 405 possible model configurations.

The relevant nitrogen line ratios for all of these (except for eight cases in which Cloudy reported a failure) are shown in Fig. 2, from which we conclude that they are consistent between the AGN and star-formation scenario, and their density dependence is largely independent of ionization parameter, metallicity or the precise shape of the incident radiation field (that is, AGN or star formation and the corresponding parameter *T*_AGN_ or t_*_).

At high densities, the NIV*λ*1483/NIV*λ*1486 ratio approaches zero (*n*_H_ ≳ 10^6^ cm^−3^), whereas Niii]*λ*1754 plateaus at a fractional contribution to the multiplet of about 0.23 at higher densities still (*n*_H_ ≳ 10^10^ cm^−3^), both pointing towards the presence of a broad line region in GN-z11 given the observed values.

**Extended Data Fig. 4:**
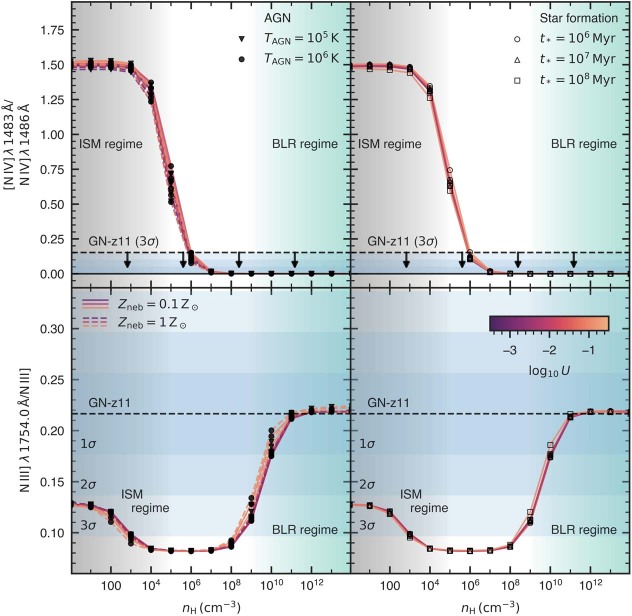
Flux ratios of density-sensitive nitrogen lines as a function of hydrogen gas density, *n*_H_. Same as Fig. 2 but where we have separated the photoionization models for AGN (left) and Star Forming galaxies (right).

Finally, to increase the readability of Fig. 2, we have separated the AGN and star-forming models in two separate panels in Extended Data Fig. 4.

### Continuum shape

If GN-z11 is a type 1 AGN, then we should be directly seeing the light from the accretion disc. In the case that the accretion disc dominates, the UV-to-optical continuum should follow a simple power law of the form *F*_*λ*_ ∝ *λ*^*β*^ with *β* = −7/3 ≈ −2.33 (ref. ^71^), as observed in type 1 AGN, and NLSy1^72,73^, modulo the UV turnover whose wavelength increases with black hole mass and also modulo effects of dust reddening, which often makes the spectrum redder.

**Extended Data Fig. 5:**
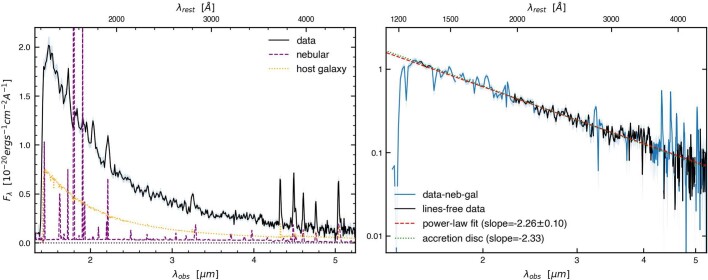
Low resolution (prism) spectrum of GN-z11. Left: Observed prism spectrum (black solid) compared with the (maximum) contribution from the host galaxy of the AGN as inferred by^8^ (orange dotted), and the nebular emission inferred from a simple Cloudy model (purple dashed) normalized to the H*γ* flux not included in the galaxy model. Right: Spectrum subtracted of the galactic and nebular continua, in a log-log scale, whose regions not affected by emission lines (solid black) have been fitted with a simple powerlaw (red-dashed) resulting into a slope of −2.26 ± 0.10, consistent with the slope expected for an accretion disc (−2.33, dotted green line).

In the case of GN-z11, the spectrum is contributed to also by the underlying galaxy identified in ref. ^8^ in the NIRCam images. This component is significantly fainter than the nuclear point-like component. It is difficult to quantitatively establish its contribution to the spectrum, because part of the light may fall outside the shutter, and in a different fraction in the four dither or pointing positions, and not easy to reconstruct because of the slight positional uncertainties discussed above. In Extended Data Fig. 5a, we show the contribution from the galactic component (dotted-orange line) to the spectrum, assuming that the entire light of the galaxy is captured by the spectrum, corresponding to about one-third of the flux and using the spectral template inferred in ref. ^8^ for the extended component.

The additional component to take into account is the nebular continuum associated with the BLR (as well as any other ionized gas in the host galaxy). The BLR typically has a low covering factor^74^, therefore the nebular continuum is not expected to be strong, but its contribution must be quantified. In most physical conditions typical of the ionized gas in the BLR, NLR or HII regions, the nebular continuum is linked to the intensity of the Balmer lines. We have estimated the nebular emission using a Cloudy model with a metallicity of 0.1 *Z*_⊙_ and a density of 10^6^ cm^−3^ (between the BLR and ISM origin scenarios) and normalized to have the same Hγ flux as observed in the spectrum of GN-z11. The nebular spectrum does not change drastically as a function of density, except obviously for the emission of the forbidden and semi-forbidden lines; however, our focus is on the nebular continuum, so we ignore the mismatch of the emission lines, as a detailed photoionization modelling of their flux is beyond the scope of this paper. We note that the nebular continuum is also included in the model spectrum fit to the extended component in ref. ^8^; therefore, not to include it twice, we have measured the Hγ flux in the ref. ^8^ spectrum and normalized the Cloudy nebular spectrum only to the Hγ flux obtained by the difference between the observed value and the flux in the ref. ^8^ model spectrum. The resulting nebular spectrum is shown with a dashed purple line in Extended Data Fig. 5a. Again, the mismatch of the emission lines should be disregarded, as the goal is not to reproduce them with the Cloudy model.

Extended Data Fig. 5b shows again the observed spectrum, in log–log scale, in which the galactic and nebular components have been subtracted. Although the noise is large, especially at long wavelengths, also as a consequence of the model subtraction procedure, the resulting spectrum is well fitted by a simple power law, in the parts not affected by the emission lines. The best-fitting slope is –2.26 ± 0.10, hence consistent with the continuum expected from an accretion disc. Note that this is not evidence in support of the presence of an AGN, as also young galaxies may have power-law shapes, it is only meant to show consistency with the AGN scenario.

We finally note that, although with a large scatter, the UV spectrum of AGN often shows a FeII hump between about 2,300 Å and about 3,100 Å (refs. ^75–79^). The prism spectrum of GN-z11 does not show an obvious FeII bump, although a more detailed analysis and modelling is required to assess the presence or absence of such a bump, which is deferred to a separate paper. However, we note that at such early epochs there is little time for the ISM to be enriched with iron through the SNIa channel^80^, so a weak or absent FeII bump would not be unexpected.

### Variability

The luminosity of AGN can be variable, from a few per cent to a factor of a few, on short (days) and long (years) timescales. We have investigated the possible presence of variability. Before the recent NIRCam images obtained in February 2023 (ref. ^8^), deep photometric observations were obtained with HST about 10 years earlier^81,82^, corresponding to about 1 year in the rest frame of GN-z11. Most of the HST photometric data points have error bars that are too large to be useful for constraining variability. However, the photometric point reported in ref. ^81^ with the F160W filter has a relatively well constrained value: 150 ± 10 nJy, within an aperture of 0.35″. NIRCam does not have the same filter, however, the photometry obtained in the F150W filter can be used and transposed to the F160W filter by using the NIRSpec prism spectrum. After extracting photometry from a 0.35″ aperture (as in ref. ^81^), and extrapolating with the NIRSpec spectrum, we obtain a F160W equivalent photometry of 141 ± 2 nJy, which is consistent with the HST previous photometry within 1*σ*. If we consider that about 30% of the flux is diluted by the host galaxy, the comparison of the photometry between the two epochs would indicate a variability of 10% at only 1*σ*. This is certainly not a detection of variability, but it is consistent with the range of variability amplitudes observed in NLSy1 and, more broadly, in type 1 AGN^83^.

### X-ray emission

GN-z11 is not detected in X-rays. Flux limits are obtained from the Chandra Deep Field North, which was a 2 Ms observation performed in 2002 (see^84^ for final results). Their sensitivity map gives a point source limit in the soft (0.5–2 keV), hard (2–7 keV) and full (0.5–7 keV) bands of 1.54 × 10^−17^, 7.9 × 10^−17^ erg cm^−2^ s^−1^ and 4.9 × 10^−17^ erg cm^−2^ s^−1^. Source detection requires a no-source probability *P* < 0.004. The tightest limit in the soft band translates to a rest frame 5.8–23.2 keV luminosity limit at *z* = 10.6 of 2.2 × 10^43^ erg s^−1^. Assuming a typical NLS1 photon index of 2.3 means that *L*_X_ (2–10 keV) is less than 3 × 10^43^ erg s^−1^.

The bolometric correction for NLS1 in the 2–10 keV band, BC_X_, is about 100 (ref. ^85^). There is a significant systematic uncertainty here due to the unseen flux in the FUV, in which the emission is expected to peak (see fig. 3 in ref. ^86^). Moreover, the 2–10 keV flux entirely originates from the corona, the early development of which and possible dependence on black hole spin are unknown (ref. ^87^ cautions against using his X-ray BC values for NLS1). Proceeding with BC_X_ = 100 means that the Chandra upper limit is almost three times above the luminosity inferred from the JWST flux at 1,400 Å. We predict a conservative SB flux of 5 × 10^−18^ erg cm^−2^ s^−1^. This would be detectable in about 1 Ms with the candidate NASA Probe mission AXIS. The coronal emission from local NLS1s is highly variable and the above BC represents a mean value (note that the intrinsic disc flux seen in the UV is much less variable^86^).

### Black hole mass estimate

For the vast majority of high redshift AGN, the black hole masses are inferred using single-epoch measurements and the so-called virial relations, that is, relations between the black hole mass, the width of the lines of the BLR and the continuum or line luminosity^37,88–94^. These relations are calibrated on nearby AGN, using either reverberation mapping techniques and/or direct dynamical measurements of the black hole. The black hole mass scales about as the square power of the width of the BLR lines and about as the square root power of the luminosity, with a proportionality constant that depends on the specific waveband (or line) for the luminosity estimation.

The most accurate virial relations would be those using Hα and Hβ. In our case, Hγ could be used as a proxy. However, as discussed, the Balmer lines are probably contributed to by the star formation in the host galaxy, hence not reliable to trace the black hole mass.

The CIII] doublet is also sometimes used to infer the black hole mass. However, this is not well resolved and, as for the case of the Balmer lines, this is probably contaminated by the ISM and star formation in the host galaxy.

MgII is often used. In our case, the MgII doublet is clearly detected, but the S/N is fairly low for the measurement of the width (Extended Data Fig. 1). If we take the width resulting from the fit and the relation provided in ref. ^95^:

$$\log \left(\frac{{M}_{{\rm{BH}}}}{{M}_{\odot }}\right)=6.86+0.5\log \left(\frac{{(\lambda {L}_{\lambda })}_{\mathrm{3,000}\mathring{\rm A} }}{1{0}^{44}{\rm{erg}}\,{{\rm{s}}}^{-1}}\right)+2\log \left(\frac{{{\rm{FWHM}}}_{{\rm{MgII}}}}{1{0}^{3}{\rm{km}}\,{{\rm{s}}}^{-1}}\right)$$

then we get a black hole mass of 1.4 × 10^6^*M*_⊙_. However, given the low S/N on the MgII doublet, we prefer to use as representative width of the BLR lines the profile of the high S/N and isolated NIV line. If we adopt this width into the equation above, we obtain a black hole mass of 1.6 × 10^6^*M*_⊙_. The uncertainty is totally dominated by the scatter in the virial scaling relation, which is about 0.3 dex (ref. ^96^).

Moreover, there are various other systematic uncertainties and caveats that can affect the black hole mass estimate. To begin with, it is not obvious that the local virial relations apply at high redshift. The main issue is whether the dependence of the BLR radius on luminosity evolves with redshift or not. The most plausible scenario is that the square root dependence of the BLR radius from luminosity is primarily set by the dust sublimation radius. In ref. ^97^, the authors argue that, given the extremely high densities in the nuclear region of AGN (hence high optical thickness even at very low dust-to-gas ratios), unless the nuclear region is totally devoid of dust, the same *R*_BL_–*L* relation is unlikely to evolve with redshift. Assessing whether the virial relations depend on the accretion rate or not is more problematic. On the one hand, in ref. ^98^, the authors argue that the effect of radiation pressure is to reduce the effective gravitational force on the clouds of the BLR; the net result is that the standard virial relations applied to BHs accreting close to the Eddington rate could underestimate the black hole mass by a factor of several. On the other hand, reverberation mapping of AGN accreting at super-Eddington has revealed that in these cases the size of the BLR is a factor of several, and up to an order of magnitude, smaller than expected from the *R*_BL_–*L* relation for sub-Eddington AGN (ref. ^99^ and references therein), which would imply that the standard virial relations overestimate, by a factor of several, the black hole masses in AGN accreting at super-Eddington. Overall, it is possible that the radiation pressure effect and the offset from the *R*_BLR_–*L* relation might cancel each other out. However, currently it is not really possible to provide an accurate assessment on how much AGN accreting at or beyond the Eddington rate might deviate from the standard virial relations.

Finally, the black hole masses from other JWST studies at *z* ≈ 4–8 (refs. ^30,93,97,100,101^) are shown in Fig. 4. These are based on the Hα or Hβ width and flux. We clarify that these are re-estimated by using the same calibrations used in ref. ^36^ for local galaxies.

### AGN bolometric luminosity estimate

We derive the bolometric luminosity of the AGN by using the continuum luminosity at *λ*_rest_ = 1,400 Å and the luminosity-dependent bolometric correction given in ref. ^87^:

$$\frac{{L}_{{\rm{b}}{\rm{o}}{\rm{l}}}}{{(\lambda {L}_{\lambda })}_{1,400\mathring{{\rm{A}}}}}=7{\left(\frac{{(\lambda {L}_{\lambda })}_{1,400\mathring{{\rm{A}}}}}{1{0}^{42}{\rm{e}}{\rm{r}}{\rm{g}}{{\rm{s}}}^{-1}}\right)}^{-0.1}$$

We also assume, as discussed in the previous sections, that 30% of the continuum flux at this wavelength is because of the underlying galactic component^8^ and that, therefore, the AGN continuum luminosity at this wavelength is 0.7 of the observed value. We infer a bolometric luminosity of 1.08 × 10^45^ erg s^−1^. The resulting ratio between bolometric and Eddington luminosity is 5.5, also affected by an uncertainty of a factor of at least 2, coming from the uncertainty on the black hole mass.

### Comparison with cosmological and hydrodynamical simulations

There is a vast literature discussing the formation of early black holes and on how they evolve in the first thousand million years, by using hydrodynamical and cosmological simulations, as well as semi-analytical models. The production and elaboration of models in this area have recently seen surge with the goal of specifically interpreting the results from JWST. It is beyond the scope of this paper to provide an exhaustive description of the assumptions and results of the several models and simulations. However, in this section, we briefly discuss that many of them can explain the properties of GN-z11 and provide some possible constraints on the seeding scenarios.

We start by considering the results obtained in ref. ^7^ from the FABLE hydrodynamical, cosmological simulation, in which they focused on the largest halo at *z* = 6 (with a virial mass *M*_200_ = 6.9 × 10^12^*M*_⊙_ of the Millennium box). The latter may appear an extreme choice; however, we note that GN-z11 does live in an overdense region and probably at the core of a protocluster^8,102^. In the FABLE simulation, the black hole seed has a mass of 10^5^*M*_⊙_ at *z* = 13. The accretion rate is capped to Eddington and uses the Bondi–Hoyle–Littleton-based formalism; however, as small scale, non-isotropic accretion is unresolved in the simulation, FABLE, like Illustris, uses a Bondi–Hoyle–Littleton rate boosted by a factor of 100. Feedback energy in FABLE scales as 10% of the available accretion energy, $\dot{E}={\epsilon }\dot{M}{c}^{2}$, where *ϵ* = 0.1 is the radiative efficiency of the accretion flow. At high redshifts, this is primarily injected as thermal energy in the vicinity of the black hole, with a duty cycle of 25 Myr. We overplot the fiducial model in ref. ^7^ in Fig. 3 (orange solid line, labelled as B23), illustrating that this can easily reproduce the mass of the black hole in GN-z11 at *z* = 10.6.

To explain the most massive BHs observed at *z* ≈ 6–7, the same study as above ^7^ also explores the scenario of earlier seeding (*z* = 18) and allows the black hole to accrete at up to two times the Eddington limit; in this case, the model could explain a black hole nearly five times more massive than GN-z11 at *z* = 10.6.

In ref. ^35^, the authors explored the early evolution of black holes using the TRINITY cosmological empirical model^103^, which is based on halo statistics from N-body simulations and incorporating empirical galactic scaling relations. The authors specifically explore the case of GN-z11. They illustrate that its mass and black hole to stellar mass ratio can be explained by their model starting with an intermediate mass seed of a few times 10^3^ seeded at *z* = 15, accreting on average at sub-Eddington rates, but intermittently also at super-Eddington. Their track is shown with a solid-teal line in Fig. 3 (labelled as Z23).

Recently, in ref. ^25^, the authors have explored the properties of GN-z11 within the context of the semi-analytical model CAT. They find that the black hole mass of GN-z11 and its location on the *M*_BH_–*M*_star_ diagram can be interpreted both in terms of light seeds (at *z* = 20–23) that can have super-Eddington accretion phases, or Eddington-limited heavy seeds formed at *z* = 14–16. Out of their various tracks, Fig. 3 shows only two samples of their tracks, in the case of a light (red-solid) and a heavy seed (red-dashed), which can both reproduce the mass of GN-z11 at *z* = 10.6 (labelled as S23). In both cases, the semi-analytical model can also reproduce the black hole to stellar mass observed in GN-z11.

In ref. ^3^, the authors suggested that the detectability of accreting BHs at high redshift by JWST implies that these are probably originating from heavy seeds. Specifically, their models can reproduce the mass of GN-z11 at *z* = 10.6 but only with seeds that are several times 10^5^*M*_⊙_, already in place before *z* = 14. GN-z11 would fall in this category, and the tracks obtained in ref. ^3^ would also explain the black hole to stellar mass ratio observed in GN-z11.

Other studies have proposed other scenarios, visualizing different seeding mechanisms, at different redshifts, and with different assumptions about the accretion and merging rates, and which are capable of reproducing the black hole mass of GN-z11 by *z* = 10.6, and generally also its black hole to stellar mass ratio^4,26,27,104^.

In sum, the properties of the black hole in GN-z11 can be explained using different assumptions, which can be broadly grouped in heavy seeds accreting at sub-Eddington rates, or intermediate–light seeds experiencing super-Eddington phases and/or modelled with a boosted Bondi accretion.

More statistics on objects such GN-z11 are required to discriminate between different scenarios. For the time being, to our knowledge, GN-z11 remains the most luminous object at *z* > 10 in all HST Deep fields (including CANDLES and Frontier Fields). It is hoped that JWST observations on larger areas (for example, in Cosmos-WEB) will find more AGN at *z* > 10 similar to GN-z11. For the time being, as discussed in the text, it is interesting to note that models and simulations were expecting a few accreting black holes with masses in the range 10^6^–10^7^*M*_⊙_ at 10 < *z* < 11 in the JADES Medium-Deep survey in the GOODS fields^3,105^. Therefore, the discovery of a 2 × 10^6^*M*_⊙_ black hole in GN-z11 is not unexpected, and a few more might be present (probably accreting at a lower rate) in the GOODS fields.

### GN-z11 and its large-scale environment

We have shown that the high nitrogen enrichment of GN-z11 is probably restricted to the BLR, whose small mass and compact size has probably undergone very rapid chemical enrichment, requiring only a few SNe.

We note that the high chemical enrichment of GN-z11 is not in contrast with the recent claim of pristine gas in the halo of GN-z11^44^. These claims are on totally different scales, with the pristine gas found several kpc away from GN-z11, whereas the high chemical enrichment is estimated to be in the nucleus of GN-z11. Regarding the claim of pristine gas in the halo of GN-z11, models expect that high-*z* massive galaxies may host pockets of pristine gas in their haloes, even down to *z* ≈ 3 (refs. ^106,107^).

## Online content

Any methods, additional references, Nature Portfolio reporting summaries, source data, extended data, supplementary information, acknowledgements, peer review information; details of author contributions and competing interests; and statements of data and code availability are available at 10.1038/s41586-024-07052-5.

### Supplementary information

Figure 1 data

Figure 2 data

## Figures and Tables

**Extended Data Table 1:**
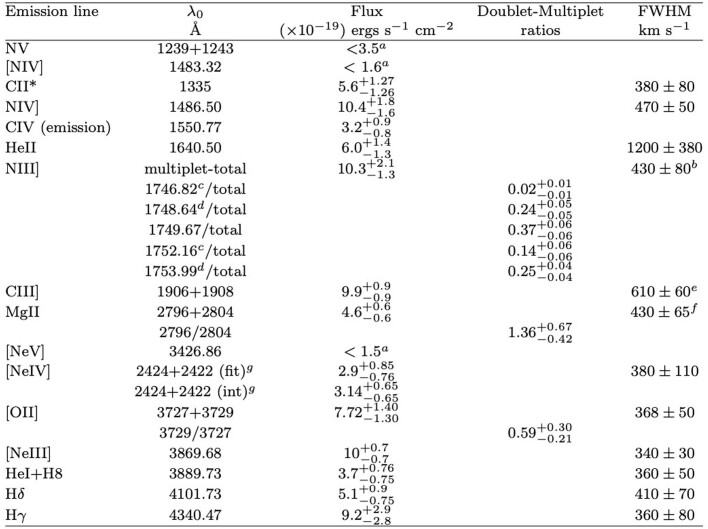
Measured fluxes and FWHM of the emission lines used in this work

Notes: ^*a*^3*σ* upper limit (in the case of NV is the upper limit on the sum of the doublet); ^*b*^the four components of the multiplet are forced to have the same width in the fit; ^*c*^as the components *λ*1746.82 and *λ*1752.16 come from the same level, their fluxes are forced to have the ratio given by their Einstein’s coefficients, i.e. 1746.82/1752.16=0.146; ^*d*^as the components *λ*1748.64 and *λ*1753.99 come from the same level, their fluxes are forced to have the ratio given by their Einstein’s coefficients, i.e. 1753.99/1748.64=1.05; ^*e*^for the CIII] doublet is totally blended and is here fitted with a single Gaussian (see text for a more complex, but uncertain decomposition); ^*f*^the two components of the MgII doublet are forced to have the same width; ^*g*^in the case of the [NeIV] doublet, as it is a key line, we provide both the result from MCMC fitting, assuming a single Gaussian (first line) and also by simply taking the integrated flux (second line).

**Extended Data Table 2:**
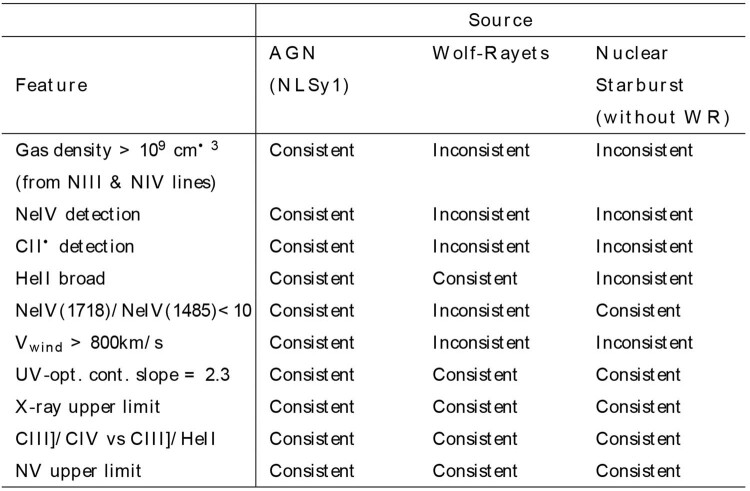
Features observed in GN-z11 versus possible energy sources

## Data Availability

The electronic version of the processed data used to produce the figures (including the 1D and 2D spectra of GN-z11) is available at the JADAES web site (jades-survey.github.io/). The NIRSpec raw data can be accessed at the JWST archive (archive.stsci.edu).
