# Author Correction: A small and vigorous black hole in the early Universe

**DOI:** 10.1038/s41586-024-07494-x

**Published:** 2024-05-17

**Authors:** Roberto Maiolino, Jan Scholtz, Joris Witstok, Stefano Carniani, Francesco D’Eugenio, Anna de Graaff, Hannah Übler, Sandro Tacchella, Emma Curtis-Lake, Santiago Arribas, Andrew Bunker, Stéphane Charlot, Jacopo Chevallard, Mirko Curti, Tobias J. Looser, Michael V. Maseda, Timothy D. Rawle, Bruno Rodríguez del Pino, Chris J. Willott, Eiichi Egami, Daniel J. Eisenstein, Kevin N. Hainline, Brant Robertson, Christina C. Williams, Christopher N. A. Willmer, William M. Baker, Kristan Boyett, Christa DeCoursey, Andrew C. Fabian, Jakob M. Helton, Zhiyuan Ji, Gareth C. Jones, Nimisha Kumari, Nicolas Laporte, Erica J. Nelson, Michele Perna, Lester Sandles, Irene Shivaei, Fengwu Sun

**Affiliations:** 1 Kavli Institute for Cosmology, University of Cambridge, Cambridge, UK; 2 Cavendish Laboratory - Astrophysics Group, University of Cambridge, Cambridge, UK; 3 Department of Physics and Astronomy, University College London, London, UK; 4 Scuola Normale Superiore, Pisa, Italy; 5 Max-Planck-Institut für Astronomie, Heidelberg, Germany; 6 Centre for Astrophysics Research, Department of Physics, Astronomy and Mathematics, University of Hertfordshire, Hatfield, UK; 7 Centro de Astrobiología (CAB), CSIC–INTA, Madrid, Spain; 8 Department of Physics, University of Oxford, Oxford, UK; 9 Sorbonne Université, CNRS, Paris, France; 10 European Southern Observatory, Garching, Germany; 11 Department of Astronomy, University of Wisconsin-Madison, Madison, WI USA; 12 European Space Agency, Space Telescope Science Institute, Baltimore, MD USA; 13 NRC Herzberg, Victoria, British Columbia Canada; 14 Steward Observatory University of Arizona, Tucson, AZ USA; 15 Center for Astrophysics - Harvard & Smithsonian, Cambridge, MA USA; 16 Department of Astronomy and Astrophysics, University of California, Santa Cruz, Santa Cruz, CA USA; 17 NSF’s National Optical-Infrared Astronomy Research Laboratory, Tucson, AZ USA; 18 School of Physics, University of Melbourne, Parkville, Victoria Australia; 19 ARC Centre of Excellence for All Sky Astrophysics in 3 Dimensions (ASTRO 3D), Melbourne, Victoria Australia; 20 Institute of Astronomy, University of Cambridge, Cambridge, UK; 21 AURA for European Space Agency, Space Telescope Science Institute, Baltimore, MD USA; 22 Department for Astrophysical and Planetary Science, University of Colorado, Boulder, CO USA

**Keywords:** Early universe, Galaxies and clusters

Correction to: *Nature* 10.1038/s41586-024-07052-5 Published online 17 January 2024

In the version of the article initially published, the coloured lines in Fig. 1b were incorrect and did not show the fit to the data. The original and corrected panel can be seen below in Fig. 1. In addition, the dotted line in panel d representing the most extreme case of low-metallicity, young star-forming galaxy should have been orange, not black. The errors have been corrected in the HTML and PDF versions of the article.

**Fig. 1:**
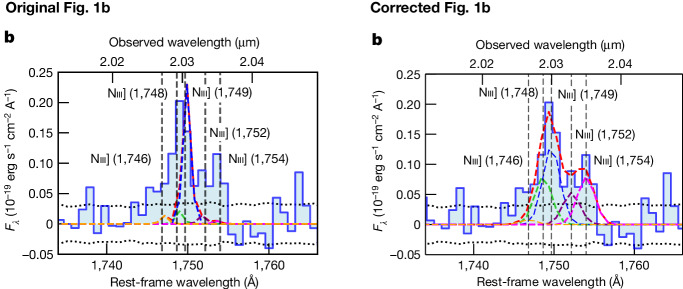
Original and corrected Fig. 1b .

